# Effects of Drought Stress on the Morphological Structure and Flower Organ Physiological Characteristics of *Camellia oleifera* Flower Buds

**DOI:** 10.3390/plants12132585

**Published:** 2023-07-07

**Authors:** Pu-Rui Guo, Ling-Li Wu, Ying Wang, Dan Liu, Jian-An Li

**Affiliations:** 1 Key Laboratory of Cultivation and Protection for Non-Wood Forest Trees of Ministry of Education, Central South University of Forestry and Technology, Changsha 410004, China; guopurui1240@163.com (P.-R.G.); wy9797up@163.com (Y.W.); 18373171797@163.com (D.L.); 2 Key Laboratory of Non-Wood Forest Products of Forestry Ministry, Central South University of Forestry and Technology, Changsha 410004, China; 3 Engineering Technology Research Center, Southern Hilly and Mountainous Ecological Non-Wood Forest Industry of Hunan Province, Changsha 410004, China; 4 Camellia Oil Tree Research Institute, Central South University of Forestry and Technology, Changsha 410004, China; 5 The Belt and Road International Union Research Center, Tropical Arid Non-wood Forest in Hunan Province, Changsha 410004, China

**Keywords:** *Camellia oleifera*, drought stress, anatomical structure, antioxidant enzyme, flower organs, hormone content

## Abstract

Investigations on the impact of drought stress on the reproductive growth of *C. oleifera* have been relatively limited compared to the extensive research conducted on its nutritional growth. To study the effects of drought stress on the growth and development of *C. oleifera* flower buds, we investigated the effects of drought stress on the bud anatomical structure, relative water content, relative electrical conductivity, antioxidant enzyme activity, osmoregulation substance content, and hormone contents of *C. oleifera* using 4-year-old potted plants (‘Huaxin’ cultivar) as experimental materials. We observed *C. oleifera* flower bud shrinkage, faded pollen colour, shortened style length, decreased relative water content, increased relative electrical conductivity, and decreased pollen germination rate under drought stress. As the stress treatment duration increased, the malondialdehyde (MDA), soluble sugar (SS), soluble protein (SP), and proline (Pro) contents, as well as peroxidase (POD), superoxide dismutase (SOD), and catalase (CAT) activities increased. Moreover, the levels of the plant hormones indole acetic acid (IAA) and cytokinin (CTK) increased, whereas those of salicylic acid (SA) and jasmonic acid (JA) decreased, and those of abscisic acid (ABA) and gibberellin a_3_ (GA_3_) first increased and then decreased. Compared to the control group, the drought treatment group exhibited stronger antioxidant capacity, water regulation ability, and drought stress protection. These results indicate that *C. oleifera* is adaptable to drought-prone environments. The results of this study provide a theoretical basis for the evaluation of drought resistance in *C. oleifera*, as well as the development of water management strategies for cultivation.

## 1. Introduction

Recent increases in the frequency of extreme weather events, including droughts, have raised concerns about the ability of plants to cope with the effects of a changing climate [1]. Drought stress affects plant growth and development and limits their geographical distribution [2]. Exposure to drought stress can damage plant cell membranes, causing electrolyte leakage that disrupts plant osmoregulation and inhibits cell division and protein synthesis through the disruption of cell metabolism. In response to drought stress, plants undergo changes in morphology and anatomical structure, forming a drought resistance response defence system by regulating metabolic mechanisms, including biofilm formation, osmosis, and protective enzyme activity [3,4,5]. For example, soluble sugar (SS), proline (Pro), and malondialdehyde (MDA) contents, as well as relative conductivity and superoxide dismutase (SOD) activity, were found to increase in the leaves of the Japanese camellia (*Camellia japonica* L.) under drought stress, indicating that this species adapts to drought stress by regulating various metabolic pathways [6].

**Figure 1:**
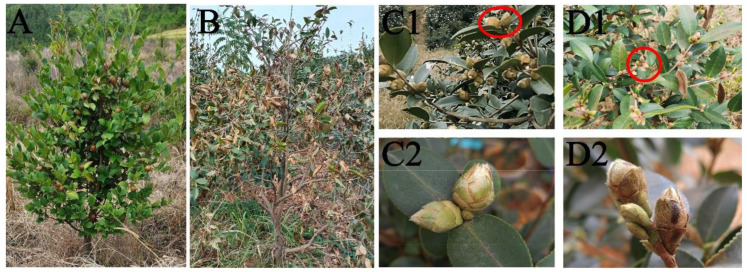
The growth status of *C. oleifera* under natural conditions and drought stress. (**A**) the growth of *C. oleifera* under natural conditions in the field; (**B**) the growth of *C. oleifera* under natural drought stress in the field; (**C1**,**C2**) the flower buds of *C. oleifera* can grow and develop under normal water supply in the field; (**D1**,**D2**) the flower buds of *C. oleifera* gradually dry up but the leaves grow normally under drought stress in the same field; (**C2**,**D2**) are marked in red circles in (**C1**,**D1**) separately.

The tea oil tree (*Camellia oleifera* Abel) is a small evergreen tree or shrub belonging to the tea family (Theaceae) (Figure 1A). Its seeds provide an edible oil that contains abundant amounts of unsaturated fatty acids (90%), oleic acid (80%), and several other nutritious substances [7,8]. *Camellia oleifera* is mainly distributed in hilly areas of southern China, mainly in Hunan, Jiangxi, and Guangxi Provinces [9]. Its flower bud differentiation is typically initiated from late May to early June and flowering occurs from mid-to late October or early November (Figure 1(C1,C2)). Because bud differentiation and flowering occur in hot summers, drought stress during this period can severely damage *C. oleifera* yield [10]. In spring 2010, sustained drought stress in southwestern China caused a *C. oleifera* disaster covering 43,700 ha, affecting 112.82 million seedlings and resulting in economic losses of RMB 366 million (Figure 1B). Therefore, studying the effects of drought stress on the growth and development of *C. oleifera* is of great theoretical and practical importance.

Drought stress is among the main environmental factors affecting growth and development and limiting the geographical distribution of *C. oleifera*. Continuous drought stress damages the growth of plant vegetative organs by reducing the photosynthetic capacity of leaves and the nutrient transport capacity of roots. It also affects flower bud growth and development through delayed flowering time, reduced pollen germination rate, and prolonged fluorescence, leading to a decrease in yield in the following year [11,12]. Persistent non-lethal drought stress can cause flower buds to gradually dry up and fall off [13]. For example, our summer 2022 field survey of *C. oleifera* in Hunan province showed that long-term high temperatures and dry weather quickly caused flower bud withering and shedding, whereas leaves were less affected and did not exhibit significant withering or death (Figure 1(D1,D2)). These results suggest a greater impact of drought stress on reproductive growth than on nutritional organs such as leaves in *C. oleifera*. Studies on the effects of drought stress on *C. oleifera* have focused on the physiological and biochemical responses of *C. oleifera* leaves to drought stress [14,15]. However, few studies have examined the effects of drought stress on *C. oleifera* flower buds or flower organs. Therefore, we investigated the effects of drought stress on the morphological structure of flower buds and physiological characteristics of floral organs in 4-year-old seedlings of one *C. oleifera* cultivar (‘Huaxin’) under simulated drought conditions using pot experiments. The results will provide important reference data for the implementation of cultivation management techniques and the evaluation and promotion of drought-resistant *C. oleifera* cultivars. 

## 2. Results

### 2.1. Effects of Drought Stress on C. oleifera Flower Bud Growth and Development

**Figure 2:**
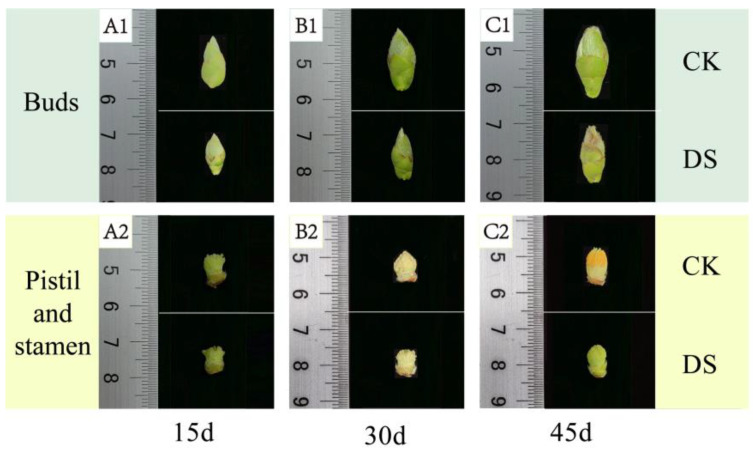
Development of *C. oleifera* flower buds under different drought stresses. (**A1**,**A2**) On the 15th day of treatment, the buds and stamens of *C. oleifera* under drought stress and in the control group; (**B1**,**B2**) On the 30th day of treatment, the buds and stamens of *C. oleifera* under drought stress and in the control group; (**C1**,**C2**) On the 45th day of treatment, the buds and stamens of *C. oleifera* under drought stress and in the control group.

The sizes of *C. oleifera* flower buds were compared for different drought stress treatment durations; the results showed that after 15, 30, and 45 days of drought stress, the mean longitudinal diameters of flower buds in the treatment groups were significantly shorter (*p* < 0.05), by 3.89, 2.49, and 4.93 mm, than those in the control groups, respectively. Similarly, the transverse diameters of flower buds in the treatment groups were significantly narrower (*p* < 0.05), by 1.05, 1.14, and 1.43 mm, than those in the control groups, respectively (Table 1). The flower buds in both groups were a soft green colour on day 15 (Figure 2(A1)) and darker with withered buds and sepal edges on day 30 (Figure 2(B1)). On day 45, flower buds in the control groups were dark green, and those in the treatment groups were yellow with significantly drier sepal edges, showing splitting at the tips of the flower buds and sepal edges (Figure 2(C1)). On days 15, 30, and 45, the longitudinal diameters of pistils and stamens in the treatment groups were significantly shorter (*p* < 0.05), by 1.11, 0.94, and 0.78 mm, than those in the control groups, respectively. The transverse diameters of pistils and stamens in the treatment groups were significantly narrower (*p* < 0.05), by 1.09, 1.51, and 1.63 mm than those in the control groups, respectively. In both groups, stamens were pale green on day 15 (Figure 2(A2)) and yellow on day 30 (Figure 2(B2)). On day 45, anthers in the control groups had turned orange, and stamens gradually matured, whereas anthers in the treatment groups were yellow-green with a significant difference in colour from the control groups (Figure 2(C2)).

### 2.2. Effects of Drought Stress on the Anatomical Structure of C. oleifera Flower Buds

**Figure 3:**
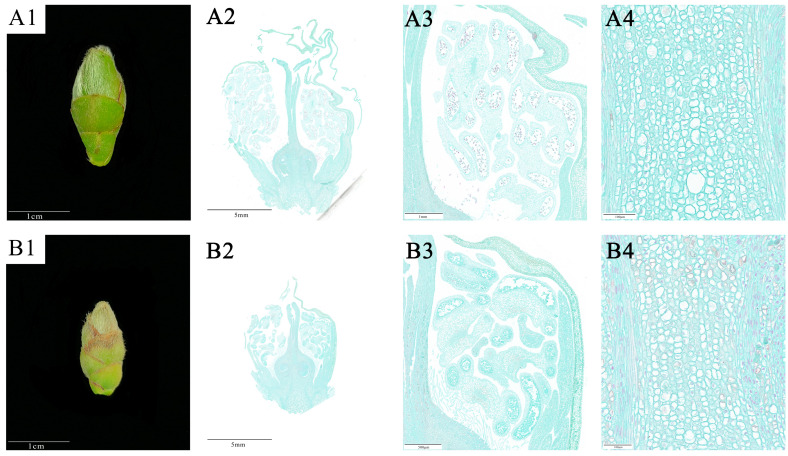
Effects of drought stress on the anatomical structure of *C. oleifera* flower buds. (**A1**–**A3**) the development of the whole flower bud and stamen group of *C. oleifera* in the control group on the 45th day of drought stress; (**B1**–**B3**) the development of the whole flower bud and stamen group of *C. oleifera* in the treatment group on the 45th day of drought stress; (**A4**,**B4**) effects of drought stress on the shape, size, and arrangement of ovarian cells in the control and treatment groups of *C. oleifera*. (**A3**), (**A4**), (**B3**), and (**B4**) are enlarged views of the local areas of (**A2**) and (**B2**).

The effects of drought stress on *C. oleifera* flower buds were mainly characterised by significant decreases in their size and length in the treatment groups on day 45 (Figure 3). To observe the changes in the internal anatomical structure of flower buds, the results of paraffin sectioning showed that drought stress resulted in reduced growth rates of the stigma, style and ovaries, and reduced numbers of pollen grains. Compared to the control group, the *C. oleifera* flower buds in the treatment groups had a smaller stigma, and shorter and thinner styles (Figure 3(A2,B2)). Moreover, on day 45 of drought stress treatment, the numbers of pollen grains were significantly reduced and the pollen grains exhibited poor development in the treatment groups (Figure 3(A3,B3)). These observations indicated a negative impact of drought stress on pollen development, leading to a decline in both quantity and quality. Furthermore, the ovary cells in the treatment groups became smaller and more tightly packed, with reduced intercellular spaces (Figure 3(A4,B4)); this finding suggests that drought stress may impede the transport of water and nutrients, thereby compromising ovule development.

### 2.3. Effect of Drought Stress on Pollen Germination of C. oleifera

**Figure 4:**
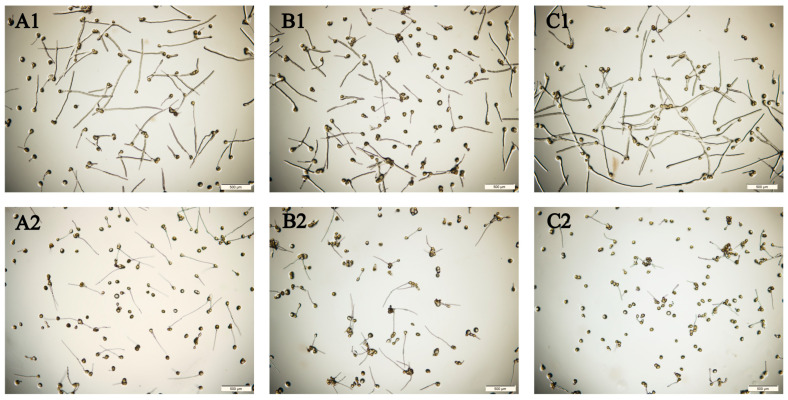
Effects of different drought treatments on the pollen germination rate of *C. oleifera*. Pollen germination rate in the control and treatment groups, respectively, at (**A1**,**A2**) day 15, (**B1**,**B2**) day 30, and (**C1**,**C2**) day 45.

Pollen germination is the key factor affecting the pollination, fertilisation, and fruit-setting rate of *C. oleifera*. Drought stress significantly reduced the pollen germination rate of *C. oleifera* (Table 2); 15, 30, and 45 days of drought stress treatment significantly decreased the pollen germination rate by 35.78% (Figure 4(A1,A2)), 47.2% (Figure 4(B1,B2)), and 61.07% (Figure 4(C1,C2)) compared with the control group, respectively (*p* < 0.05). The pollen germination rate also decreased significantly under drought stress with the prolongation of treatment (*p* < 0.05). These results indicate that drought stress significantly inhibits pollen development in *C. oleifera* and that pollen germination rate decreases as treatment duration increases.

### 2.4. Effects of Drought Stress on the Relative Water Content, Relative Electrical Conductivity, and MDA Content in C. oleifera Petals

**Figure 5:**
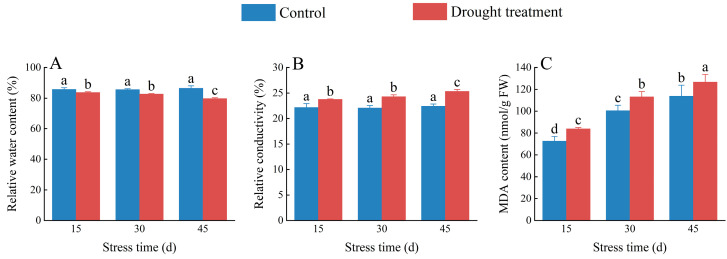
Effects of drought stress on the relative water content (**A**), relative conductivity (**B**), and malondialdehyde (MDA) (**C**) content in *C. oleifera* petals. Bars indicate standard error. Different letters indicate significant differences among groups (*p* < 0.05).

Relative water content and relative conductivity directly reflect the degree of water deficit in plants under drought stress and are commonly used to measure a plant’s water retention capacity. The relative water content of *C. oleifera* petals decreased gradually as the duration of drought stress increased (Figure 5A); however, there was no significant difference among control groups on day 15, 30, or 45 (*p* > 0.05). There was no significant difference in the relative water content of *C. oleifera* petals between the treatment groups on day 15 or 30 (*p* > 0.05), whereas, on day 45, the treatment group had a significantly lower (6.72%) relative water content than the control group (*p* < 0.05; Tables S2 and S3). The relative electrical conductivity of *C. oleifera* petals increased with the duration of drought stress (Tables S4 and S5), with no significant difference among treatment groups on day 15 or 30 (*p* > 0.05). However, after 45 days, relative electrical conductivity was significantly higher (13.16%) in *C. oleifera* petals in the treatment group than in the control group (Figure 5B). The MDA content of *C. oleifera* petals was significantly higher under drought stress, by 15.37%, 12.77%, and 11.26% than in the control group on days 15, 30, and 45, respectively (Figure 5C), and MDA content increased gradually with treatment duration. However, the relative increase rate of MDA content decreased as treatment duration increased (Tables S6 and S7).

### 2.5. Effects of Drought Stress on Antioxidant Enzyme Activity and Osmoregulation Substance Content in C. oleifera Pistils and Stamens

**Figure 6:**
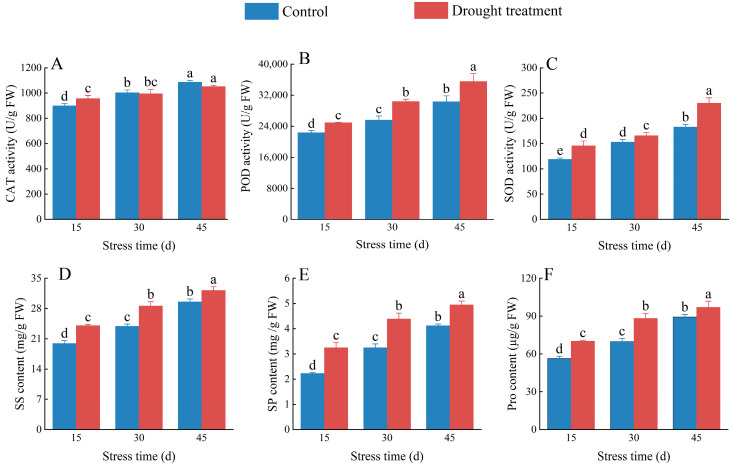
Effects of drought stress on catalase (CAT) (**A**), peroxidase (POD) (**B**), and superoxide dismutase (SOD) (**C**), activity and soluble sugar (SS) (**D**), soluble protein (SP) (**E**), and proline (Pro) (**F**), content in *C. oleifera*. Bars indicate standard error. Different letters indicate significant differences among groups (*p* < 0.05).

The CAT activity of *C. oleifera* pistils and stamens increased significantly by 6.38% compared to the control under drought stress after 15 days of drought stress treatment (*p* < 0.05). CAT activity gradually increased with exposure time in both the treatment and control groups (Figure 6A; Tables S8 and S9). POD activity in *C. oleifera* pistils and stamens under drought stress increased significantly by 11.49%, 18.78%, and 17.26%, respectively, compared to the control (*p* < 0.05; Figure 6B; Tables S10 and S11), and increased gradually with the extension of drought stress duration. After 15, 30, and 45 days of drought stress, SOD activity in *C. oleifera* pistils and stamens increased significantly by 18.23%, 7.74%, and 20.52%, respectively, compared to the control group (*p* < 0.05); SOD activity generally increased as treatment duration increased (Figure 6C; Tables S12 and S13). These results indicate that *C. oleifera* mounted an antioxidant response to mitigate oxidative damage resulting from drought stress.

The SS content of *C. oleifera* pistils and stamens increased significantly in both treatment and control groups over time (Tables S14 and S15); SS content in the treatment groups increased significantly by 20.60%, 19.66%, and 8.89% compared with the control after 15, 30, and 45 days, respectively (*p* < 0.05; Figure 6D). Similarly, the SP content of *C. oleifera* pistils and stamens increased significantly by 45.78%, 34.96%, and 20.07% compared with the control after 15, 30, and 45 days, respectively (Figure 6E), and both groups showed a gradual increase over time (Tables S16 and S17). The Pro content of *C. oleifera* pistils and stamens also increased significantly under drought stress compared with the control (*p* < 0.05; Tables S16 and S17), with increases of 24.40%, 26.49%, and 8.72% compared with the control after 15, 30, and 45 days, respectively, and a gradual increase in both groups as exposure time increased (Figure 6F). These results indicate that the response mechanism of *C. oleifera* to drought stress is mainly to reduce or delay the occurrence of oxidative damage by increasing antioxidant enzyme activity.

### 2.6. Effects of Drought Stress on Endogenous Hormone Content in C. oleifera

**Figure 7:**
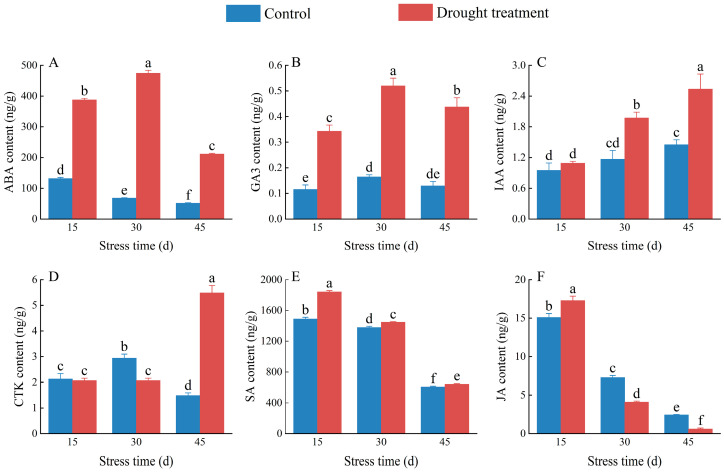
Effects of drought treatment on abscisic acid (ABA) (**A**), gibberellin (GA_3_) (**B**), indole acetic acid (IAA) (**C**), cytokinin (CTK) (**D**), salicylic acid (SA) (**E**), and jasmonic acid (JA) (**F**) content in *C. oleifera* pistils and stamens. Bars indicate standard error. Different letters indicate significant differences among groups (*p* < 0.05).

The ABA content of *C. oleifera* pistils and stamens in the control group decreased over time, whereas that in the treatment group first increased and then decreased (Tables S20 and S21), with levels significantly higher, by 194.23%, 594.28%, and 309.45%, than those in the control group at 15, 30, and 45 days, respectively (Figure 7A; *p* < 0.05). The GA_3_ content of *C. oleifera* pistils and stamens under drought stress first increased and then decreased over time (Tables S22 and S23); compared with the control group, the GA_3_ content in the treatment group was 195.95%, 214.76%, and 237.93% higher at 15, 30, and 45 days, respectively (Figure 7B). The IAA content of *C. oleifera* pistils and stamens under drought stress increased significantly by 14.72%, 69.01%, and 74.60%, respectively, compared to the control group at 15, 30, and 45 days of treatment, respectively (Figure 7C; Tables S24 and S25; *p* < 0.05). There was no significant difference (*p* > 0.05) in CTK content in *C. oleifera* pistils and stamens between the treatment and control groups on day 15 (Figure 7D); However, on day 30 and 45, drought stress had significant effects on the CTK content compared with the control group. Specifically, on day 30, drought stress significantly decreased the CTK content by 29.74% (*p* < 0.05), while on day 45, it significantly increased the CTK content by 268.86% (*p* < 0.05). showing a decrease followed by an increase over time (Tables S26 and S27). The SA content of *C. oleifera* pistils and stamens under drought stress treatment increased significantly by 23.71%, 5.05%, and 5.66% compared to the control group on days 15, 30, and 45, respectively (Figure 7E; *p* < 0.05), showing a gradual decrease over time (Tables S28 and S29). The JA content of *C. oleifera* pistils and stamens under drought stress increased significantly by 14.42% compared to the control group on 15 days of treatment (*p* < 0.05; Figure 7F), followed by decreases of 43.48% and 74.43% on 30 and 45 days of treatment, respectively, compared to the control group (Tables S30 and S31). This finding suggests a complex response mechanism of *C. oleifera* to drought stress, with different hormones playing different roles during different response periods. 

## 3. Discussion

Flower bud development plays a critical role in plant reproductive growth processes, which are significantly affected by drought stress [16]. Drought stress reduces plant water content, leading to cellular atrophy and apoptosis, ultimately affecting flower development and growth in terms of flower size and morphology [17]. In this study, we investigated changes in the size and colour of *C. oleifera* flower buds under different drought stress durations; the results showed that the longitudinal and transverse diameters of flower buds, stamens, and pistils in drought treatment groups were significantly smaller than those in control groups, indicating that drought stress affects the growth rate and development of *C. oleifera* flower bud organs, which is consistent with the findings of previous studies [18]. We also observed that stamens and pistils in the treatment groups gradually increased in size as the duration of stress treatment increased, suggesting that *C. oleifera* adapts to long-term drought stress by exhibiting stronger stress responses. Drought stress affects the physiological metabolism of plants, leading to alterations in flower colour and morphology [19]. In this study, after 45 days of stress, sepal edges in the treatment group withered and turned yellow, and anthers turned yellow-green, producing significant differences from the control group; this finding indicates that long-term drought stress leads to incomplete anther development in this species, possibly due to long-term drought stress-induced plant growth inhibition and physiological metabolic imbalance [20].

Drought stress can also reduce the quality and quantity of pollen, thereby affecting flower pollination and seed formation, with a potential impact on plant reproduction and population survival. In this study, we assessed the pollen germination of *C. oleifera* and found that drought stress significantly impacted the pollen germination rate. As the treatment duration was extended, the pollen germination rate of *C. oleifera* decreased continuously; this, in turn, affected the pollination, fertilisation, and fruit-setting rates of *C. oleifera*. Because pollen is crucial in plant reproduction, its germination rate directly determines the success rate of plant reproduction [21]. Our results revealed a significant decrease in *C. oleifera* pollen germination rate, perhaps due to insufficient water in the plant body, which hinders the normal transport of nutrients, and can limit pollen development and germination. Therefore, drought stress has a direct negative impact on the reproductive processes of *C. oleifera*. Drought environments also have significant impacts on the anatomical and histological structures of plants. Drought stress can result in leaf thickening and vessel narrowing, and reductions in cell gaps and stomatal numbers, thereby affecting plant growth and physiological processes [22]. In this study, we observed changes in *C. oleifera* flower buds, styles, pollen grains, and ovaries under drought stress. It was found that the flower organs were stunted or even stagnant under drought stress, indicating a negative impact of drought stress on reproductive growth in *C. oleifera*, which can lead to decreased yield.

Relative water content is a critical indicator reflecting the water status of plants [23]. The results of this study showed that the relative water content of *C. oleifera* petals decreased significantly under drought stress, indicating that the petal is significantly affected by drought stress; this finding is consistent with previous studies [24]. Relative conductivity is an indicator of cell membrane integrity [25]. Our findings suggest that the relative conductivity of *C. oleifera* increases under drought stress, indicating that drought stress damages the cell membrane, which can lead to ion leakage and cytoplasm liquefaction. Membrane lipid peroxidation is a vital index of oxidative damage to plant cell membranes. MDA content reflects the degree of membrane lipid peroxidation, and its elevation aggravates damage to the cell membrane [26]. Our results revealed significantly higher MDA activity in *C. oleifera* flower buds under drought stress than in the control group, with activity increasing as the treatment duration was extended. This finding indicates that water scarcity leads to the accumulation of excessive reactive oxygen species (ROS), causing severe damage to the plant cell membrane and hindering protein synthesis, consistent with previous research [27].

Drought stress disrupts the equilibrium between the generation and scavenging of ROS in plant cells, leading to oxidative stress. When ROS accumulation surpasses a certain threshold, it causes the degradation of biological macromolecules and ruptures cell membranes, inducing and exacerbating membrane lipid peroxidation [28]. Efficient antioxidant defence systems in plants have evolved to manage ROS-induced oxidative stress. Antioxidant enzymes such as CAT, SOD, and POD play crucial roles in these systems [29]. In this study, CAT activity in *C. oleifera* under drought stress was significantly higher after 15 days of treatment compared to the control but did not differ from the control after 30 and 45 days of treatment. CAT activity showed an increasing trend throughout the treatment period, indicating that it is a key enzyme of the *C. oleifera* flower bud antioxidant defence system. CAT, SOD, and POD together eliminate free radicals from the plant body and protect it from damage due to drought stress. As drought stress treatment duration increased, excess free radicals produced by *C. oleifera* reacted with CAT, decreasing its activity compared to the control. These results are consistent with those of previous studies [30]. POD activity exhibits dynamic changes in various plant tissues, and is closely related to plant growth and development and the degree of oxidation [28]. Our results showed that *C. oleifera* POD activity was significantly higher in the drought stress treatment group than in the control group, consistent with a previous study [31]. SOD is a critical ROS-scavenging enzyme in plant cells. We observed a continuous increase in SOD activity in the treatment group as treatment duration increased, indicating a disruption in the balance between the generation and clearance of free radicals within stamen and pistil cells, resulting in ROS accumulation and damage to membrane selective permeability.

Osmoregulation substances such as SS, SP, and Pro affect plant osmotic pressure, improving their water retention ability and enabling them to resist drought stress and maintain normal cellular activity [32]. Following exposure to drought stress, plants produce osmoregulation substances to stabilise the water potential difference between the interior and exterior of cells, conferring stress tolerance [33]. SS is an osmoregulation substance that accumulates in plants under drought stress, regulating cell water potential, reducing water loss, and helping plants to adapt to drought stress [34]. We found that SS content in *C. oleifera* pistils and stamens increased significantly under drought stress over time compared to the control, suggesting that *C. oleifera* flower buds accumulate more SS to alleviate the damage caused by drought and maintain their intracellular water balance, which is consistent with the findings of previous studies [35,36]. SP is a plant osmoregulation substance and nutrient; its content reflects plant adaptability to drought stress because it enhances the water-holding capacity of cells [37,38]. Under drought stress, the SP content of *C. oleifera* pistils and stamens increased significantly over time compared to the control, suggesting that *C. oleifera* accumulated more SP to maintain intracellular water balance and improve nutrition, consistent with the results of previous studies [39,40]. Pro is a physiological indicator of drought resistance physiology in plants; it maintains plant cell integrity and stability and facilitates normal metabolic activity under adverse conditions such as drought stress. Rapid increases in Pro content have been interpreted as drought resistance cues in forest trees [41,42]. We found that the Pro content of *C. oleifera* pistils and stamens increased significantly under drought stress over time compared to the control, suggesting that *C. oleifera* accumulates Pro to maintain cell water potential, reduce water loss, and adapt to drought stress, consistent with previous findings [43].

Plant hormones are signalling molecules that allow plants to sense changes in their external environment, regulate growth, resist adverse environmental conditions, and maintain survival. In plants subjected to abiotic stresses, a series of adaptive mechanisms are triggered to reduce damage through regulation of the synthesis, transportation, and signal transduction of endogenous hormones [44]. For example, drought stress has been shown to reduce auxin content in *Festuca arundinacea* Schreb and maintain high ABA levels in *Rhododendron annae* Franch leaves [45,46]. In this study, the ABA content of *C. oleifera* flower buds increased significantly with the severity of drought stress, reaching a peak at 30 days and then decreasing. This trend may be due to the initial role of ABA as a signalling molecule that induces and activates the plant response to drought stress, while also promoting senescence and abscission, and inhibiting growth in the late stage of drought stress. Cytokinins play important regulatory roles in protein synthesis, enzyme activity, and cell metabolism, and can improve plant drought resistance [47]. Our results showed a significant increase in the content of the CTK on 45 days of drought stress treatment, suggesting that cytokinins and ABA have antagonistic effects on the pistils and stamens of *C. oleifera*. GA_3_ is involved in various stages of plant growth and development, including the promotion of cell division and elongation, root growth, and floral induction [47,48]. We observed a significant increase in the GA_3_ content of *C. oleifera* pistils and stamens under drought treatment compared to the control, suggesting that these organs mitigate the damage caused by drought by increasing GA_3_ content. Similar to ABA, IAA is a natural plant hormone that participates in various stress responses [49]. Changes in ABA content exhibit various effects in different plant species. For example, IAA content and drought severity were negatively correlated in Hippophae rhamnoides leaves under different levels of drought stress, with higher drought severity associated with lower auxin content [50]. Conversely, higher degrees of drought promoted auxin accumulation in Gossypium hirsutum [51]. Our results showed that auxin content increased significantly with drought severity, exacerbating its accumulation in pistils and stamens, promoting their growth and development, and increasing their drought resistance. SA plays key roles in biological and abiotic stress responses by participating in biological processes such as photosynthesis, transpiration, ion uptake, and transport [52]. Under abiotic stress, the SA production pathway is activated to promote damage resistance in plants. We found that SA content increased initially and then decreased with the extension of treatment duration, which was opposite to the effect observed for auxin, suggesting that these two plant hormones have antagonistic effects. JA plays a significant role in plant responses to abiotic stresses, acting as a signal molecule by regulating plant stomatal closure after signal transduction to reduce plant water loss in response to environmental signal stimuli such as low temperature, light, and water availability [53]. In this study, JA content peaked at 15 days and then decreased significantly, indicating that JA exhibits different responses at different stages of drought stress in the stamens and pistils of *C. oleifera* flower buds.

## 4. Materials and Methods

### 4.1. Plant Materials, Treatments, and Sample Collection

**Figure 8:**
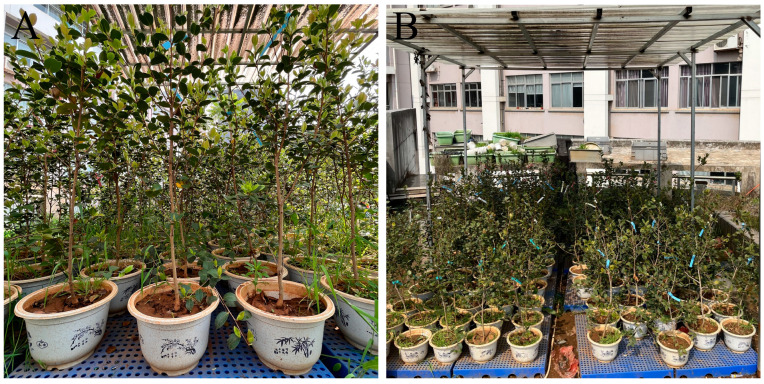
Experimental site conditions. (**A**) the 4-year-old *C. oleifera* potted dwarf plants were under identical water and fertiliser management conditions on the roof; (**B**) a canopy was built to prevent external environmental interference, and the potted dwarf plants were placed on a high of 15 cm bracket.f 15 cm.

We obtained the *C. oleifera* cultivar ‘Huaxin’ from Zhongsen Forestry Technology Co., Ltd. (Chaling County, Hunan Province, China). On 9 July 2022, healthy 4-year-old *C. oleifera* plants were transplanted into plastic pots (30 × 30 × 21 cm) containing a mixture of yellow soil, peat soil, perlite, and vermiculite (*V*:*V*:*V*:*V* = 5:2:2:1). From 10 July to 20 September 2022, the potted dwarf plants were maintained under identical water and fertiliser management conditions on the roof of the Life Science Building of Central South University of Forestry and Technology, Changsha, China (28°10′ N, 113°23′ E). A canopy was built to prevent external environmental interference, and the potted dwarf plants were placed on a shelf at a height of 15 cm (Figure 8B). The experiment began on 20 September 2022, during the experimental period, the average temperature was 34 °C, with an average relative humidity of 73%, and the relative soil water content (RSWC) of the drought stress treatment groups was maintained at 25–35% by measuring the RSWC every 4 h from 07:00 to 20:00 daily, watering when RSWC decreased below 25%; when watering was required, we re-tested the RSWC 0.5 h later. The same treatment was applied to the control groups, but with a watering threshold of 75%, to maintain an RSWC of 75–80%. The measurement of RSWC mentioned above is conducted using the SANKU SK-100 soil moisture meter from Japan.

We measured responses to drought stress at 15, 30, and 45 days of treatment. Flower organs and flower buds were collected from the potted plants at each time point at approximately 10:00. Sampling was conducted in triplicate for the treatment and control groups, where DS indicates drought stress treatment and CK indicates control. The treatment and control groups each contained 15 pots and label them separately (Table S1). Pistils and stamens were collected for physiological measurements and petals were collected to determine relative moisture content and relative conductivity; each 5-g sample had three replicates. Samples were quickly wrapped with tin foil, labelled, immersed in liquid nitrogen for 30 min for rapid freezing, and then refrigerated at −80 °C. Flower buds were collected for anatomical structure observation; each sample contained three unfolded buds.

### 4.2. Observation of Flower Bud Anatomical Structure

Flower buds collected for anatomical structural observation were peeled to remove all but two or three sepals, transferred to 70% ethanol for storage, and then fixed with Carnoy’s solution (ethanol:acetic acid = 3:1) for 12 h. After fixation and preservation, conventional paraffin sectioning was performed to observe flower bud growth and development. After material selection and repair, the fixed materials were subjected to stepwise alcohol dehydration, xylene transparency, waxing, embedding, slicing (section thickness, 4 µm), dewaxing, and sectional staining with safranin/fast green and Canadian gum sealing to create permanent slices. Typical sections were selected and imaged using a digital scanner (PANNORAMIC SCAN; 3DHistech, Budapest, Hungary); the digital images were processed using the Case Viewer software (Dentsply Sirona, Charlotte, NC, USA) for anatomical structural analysis [54].

### 4.3. Determination of Pollen Germination 

The pollen germination rate of *C. oleifera* was assessed using an agar culture method. Flower buds at full bloom were collected, and the anthers were removed using tweezers, placed on sulfuric acid paper, and exposed to a 100 W light bulb for 3–4 h at a constant temperature of 25–28 °C to disperse the pollen. Then, the pollen was immediately sown on a medium consisting of 0.01% boric acid, 10% sucrose, and 1% agar. Following 2 h of culture at 25 °C, the pollen germination rate was calculated by dividing the number of germinated pollen grains by the total number of pollen grains and multiplying the result by 100% [55].

### 4.4. Determination of Physiological Indicators

#### 4.4.1. Relative Water Content and Relative Conductivity

Relative water content was determined using the drying method. *Camellia oleifera* flower petals were weighed to obtain their fresh weight, soaked in tap water for 24 h, and re-weighed. The petals were then placed in an oven and baked at 80 °C until a constant weight was reached. The dry weight of the petals was obtained, and the relative water content and water deficit of the petals were calculated. Relative water content was calculated as (natural fresh weight—dry weight)/(saturated fresh weight—dry weight) × 100% [56].

To measure the relative conductivity of *C. oleifera* petals, we weighed 1 g of petals and rinsed them twice with deionised water. Then, we cut the petals into small pieces (length, ~1 cm) and placed them in a beaker containing 20 mL of deionised water to soak for 20 min with gentle stirring using a glass rod. Next, we measured the conductivity of the sample using an electrical conductivity meter to obtain the initial conductivity reading (R1). Then, the beaker was placed in a water bath and heated for 15 min to destroy the plant tissue. After cooling, we measured the conductivity of the sample to obtain the boiling conductivity (R2). The relative conductivity of the *C. oleifera* petals was calculated as (R1/R2) × 100% [56].

#### 4.4.2. Antioxidant Enzymes

Enzyme extracts were prepared from 0.5-g samples of *C. oleifera* stamen and pistil tissues to determine SOD, catalase (CAT), and peroxidase (POD) activity using a chilled phosphate buffer. The tissues were homogenised and centrifuged at 10,000× *g* for 20 min at 4 °C, and the resulting supernatant was used as the enzyme extract. SOD activity was determined using the nitrogen blue tetrazole method and calculated as described previously [57]. CAT activity was measured as described previously [58], with 1 enzyme activity unit (U) defined as the amount required to change the optical density at 240 nm (OD240) by 0.1 within 1 min. POD activity was determined using the guaiacol method [59], for which 1 U was determined as the amount of enzyme required to reduce the OD470 by 0.1 within 1 min. The Infinite F200 microplate reader (Tecan, Männedorf, Switzerland) was used for the determination of antioxidant enzyme activity. MDA content was determined using the thiobarbituric acid method [60].

#### 4.4.3. Osmoregulation Substances

Soluble sugar (SS) was extracted from 1 g of *C. oleifera* stamen and pistil samples in a tube and then extracted twice at 100 °C for 30 min. The filtered solution was transferred into a 25-mL volumetric flask, and the SS content was determined using the anthrone colourimetric method [61]. Soluble proteins (SP) were extracted from 1 g of *C. oleifera* stamen and pistil samples by grinding them in 3 mL of extraction buffer consisting of 25 mmol L^−1^ potassium phosphate buffer (pH 7.5), 5 mmol L^−1^ EDTA-Na2, and 5 mmol L^−1^ cysteine. The suspension was transferred to centrifuge tubes and clarified by centrifugation for 15 min at 12,000× *g* at 4 °C. The supernatant was used for SP content measurement using the Coomassie brilliant blue method [62], with bovine serum albumin as the standard protein. Pro was extracted from 1 g of *C. oleifera* stamen and pistil samples in a tube with 10 mL water at 100 °C and then extracted twice with the same volume of water at 100 °C. The filtered solution was transferred into a 25-mL volumetric flask, and the Pro content was determined using the ninhydrin colourimetric method, with leucine as the standard amino acid [63]. The experiment employed a Hitachi UV-Vis spectrophotometer (Hitachi, Beijing, China) with the model number U-331003040425.

#### 4.4.4. Endogenous Hormone Content

Liquid chromatography [64] was used to determine the levels of plant hormones, including abscisic acid (ABA), indole acetic acid (IAA), cytokinin (CTK), gibberellin (GA3), salicylic acid (SA), and jasmonic acid (JA), in *C. oleifera* stamen and pistil samples. Immediately after collection, the stamen and pistil tissues were frozen in liquid nitrogen and then subjected to ultrasonic extraction with methanol. The resulting supernatant was filtered and collected for further analysis. Sample pre-treatment involved adding the sample to 10% sulfuric acid solution, mixing it thoroughly, shaking for 30 min, adding cyclohexane for separation, and then adding methanol and boric acid solution for ultrasonic treatment and centrifugal separation to finally obtain the sample solution. A C18 reversed-phase liquid chromatographic column was used under optimised chromatographic conditions with a mobile phase consisting of formic acid acetonitrile (95:5, *v*/*v*). The detection wavelength was set at 254 nm, the column temperature was maintained at 30 °C, the flow rate was 1 mL min^−1^, and the injection volume was 20 μL. Standard curves were prepared by injecting different concentrations of ABA, IAA, IP, GA3, SA, and JA standard solutions into the chromatographic column after sample pre-treatment to record the peak area and establish a standard concentration curve. Following sample pre-treatment, the sample solution was injected into the chromatographic column, the peak area was recorded, and the content of each plant hormone was calculated using the respective standard curve. The liquid chromatography instrument used in the experiment is UPLC (Ultra Performance Liquid Chromatography), and the chromatographic column used is Agilent EC C18 with dimensions of 4.6 mm × 50 mm.

### 4.5. Analyses

All data were obtained from three or more independent replicates. The Origin 2022 software (OriginLab, Northampton, MA, USA) was used to process and plot the data, and to calculate the sample standard deviation. The SPSS 22.0 software (IBM Corp., Armonk, NY, USA) was used to conduct one-way analysis of variance to test for significant differences.

## 5. Conclusions

Drought stress significantly influences the physiological and biochemical kinetics of *C. oleifera* floral buds. Manifestations of this stress are perceived as a decrement in relative water content paired with an augmentation in relative electrical conductivity, consequently postponing, or even hindering, the maturation process of the floral buds. Additionally, the stress response triggers an elevation in MDA, osmoregulatory compounds, IAA, CTK, and the activity of antioxidant enzymes. Concurrently, it is observed that levels of SA and JA undergo reduction. The levels of ABA and GA_3_ initially demonstrate an increased concentration, which then descends under continuous drought stress. This ensemble of modifications cumulatively amplifies the adaptive physiological features of *C. oleifera*, thereby affecting its anatomical constitution, endogenous hormonal balance, osmotic regulation, and antioxidant enzymatic activity. Hence, it can be posited that drought stress plays a pivotal role in modulating the anatomical structure, endogenous hormonal landscape, osmotic balance, and antioxidant activity in *C. oleifera* floral buds, thus enhancing their drought resilience.

## Figures and Tables

**Table 1:** Effects of drought stress on the sizes of various parts of *Camellia oleifera* flower buds.

Flower Bud Part	Treatment	Longitudinal Diameter (mm)			Lateral Diameter (mm)		
		15 Days	30 Days	45 Days	15 Days	30 Days	45 Days
Bud	Control	16.48 ± 1.31 ^bc^	17.83 ± 0.87 ^b^	23.43 ± 1.64 ^a^	6.44 ± 0.13 ^d^	7.68 ± 0.13 ^c^	9.53 ± 0.30 ^a^
	Drought	12.59 ± 0.79 ^d^	15.34 ± 0.72 ^c^	17.70 ± 0.72 ^b^	5.39 ± 0.13 ^e^	6.54 ± 0.26 ^d^	8.10 ± 0.17 ^b^
Pistil/Stamen	Control	7.68 ± 0.16 ^c^	8.41 ± 0.15 ^b^	9.13 ± 0.20 ^a^	5.21 ± 0.14 ^c^	6.05 ± 0.09 ^b^	6.65 ± 0.11 ^a^
	Drought	6.57 ± 0.33 ^d^	7.47 ± 0.37 ^c^	8.35 ± 0.31 ^b^	4.12 ± 0.09 ^e^	4.54 ± 0.14 ^d^	5.02 ± 0.10 ^c^

Data are means ± standard deviation (*n* = 3). Different letters indicate significant differences among groups (*p* < 0.05).

**Table 2:** Pollination germination rates (%) of *C. oleifera* in control and drought treatment groups over time.

Treatment	Pollen Germination Rate (%)		
	15 Days	30 Days	45 Days
Control	83.08 ± 2.55 ^a^	83.24 ± 1.32 ^a^	83.79 ± 1.17 ^a^
Drought	47.30 ± 1.41 ^b^	36.04 ± 1.34 ^c^	22.72 ± 1.06 ^d^

Data are means ± standard deviation (*n* = 3). Different letters indicate significant differences between groups (*p* < 0.05).

## Data Availability

The data is contained within the article and the Supplementary Materials.

## Supplementary Materials

The following supporting information can be downloaded at: https://www.mdpi.com/article/10.3390/plants12132585/s1, Table S1. Labels for the materials in this experiment; Table S2. The effect of drought stress on the Relative Water content of *C. oleifera* petals; Table S3. The effect of drought stress on the significant difference in Relative Water content among groups of *C. oleifera* petals; Table S4. The effect of drought stress on the Relative conductivity of *C. oleifera* petals. Table S5. The effect of drought stress on the significant difference in Relative conductivity among groups of *C. oleifera* petals; Table S6. The effect of drought stress on the MDA content of *C. oleifera* petals; Table S7. The effect of drought stress on the significant difference in MDA content among groups of *C. oleifera* petals; Table S8. The effect of drought stress on the CAT activity in *C. oleifera* pistils and stamens; Table S9. The effect of drought stress on the significant difference in CAT activity among groups of *C. oleifera* pistils and stamens; Table S10. The effect of drought stress on the POD activity in *C. oleifera* pistils and stamens; Table S11. The effect of drought stress on the significant difference in POD activity among groups of *C. oleifera* pistils and stamens; Table S12. The effect of drought stress on the SOD activity in *C. oleifera* pistils and stamens; Table S13. The effect of drought stress on the significant difference in SOD activity among groups of *C. oleifera* pistils and stamens; Table S14. The effect of drought stress on the SS content in *C. oleifera* pistils and stamens; Table S15. The effect of drought stress on the significant difference in SS content among groups of *C. oleifera* pistils and stamens; Table S16. The effect of drought stress on the SP content in *C. oleifera* pistils and stamens; Table S17. The effect of drought stress on the significant difference in SP content among groups of *C. oleifera* pistils and stamens; Table S18. The effect of drought stress on the Pro content in *C. oleifera* pistils and stamens; Table S19. The effect of drought stress on the significant difference in Pro content among groups of *C. oleifera* pistils and stamens; Table S20. The effect of drought stress on the ABA content in *C. oleifera* pistils and stamens; Table S21. The effect of drought stress on the significant difference in ABA content among groups of *C. oleifera* pistils and stamens; Table S22. The effect of drought stress on the GA3 content in *C. oleifera* pistils and stamens; Table S23. The effect of drought stress on the significant difference in GA3 content among groups of *C. oleifera* pistils and stamens; Table S24. The effect of drought stress on the IAA content in *C. oleifera* pistils and stamens; Table S25. The effect of drought stress on the significant difference in IAA content among groups of *C. oleifera* pistils and stamens; Table S26. The effect of drought stress on the CTK content in *C. oleifera* pistils and stamens; Table S27. The effect of drought stress on the significant difference in CTK content among groups of *C. oleifera* pistils and stamens; Table S28. The effect of drought stress on the SA content in *C. oleifera* pistils and stamens; Table S29. The effect of drought stress on the significant difference in SA content among groups of *C. oleifera* pistils and stamens; Table S30. The effect of drought stress on the JA content in *C. oleifera* pistils and stamens; Table S31. The effect of drought stress on the significant difference in JA content among groups of *C. oleifera* pistils and stamens.
