# Supplementary material for: Effects of Drought Stress on the Morphological Structure and Flower Organ Physiological Characteristics of Camellia oleifera Flower Buds
Source: Plants (Basel). 2023 Jul 7;12(13):2585. doi: 10.3390/plants12132585 (PMC10346383; doi:10.3390/plants12132585)
Supplement: Supplementary file 1 [file plants-12-02585-s001.zip › plants-2425711-supplementary.pdf]

plants

MDPI

Supplementary Materials

Table S1. Labels for the materials in this experiment.

Cultivar	Drought stress time	Label
'Huaxin'	15d	HX-15d-CK1 to HX-15d CK3
		HX-15d-DS1 to HX-15d DS3
	30d	HX-30d-CK1 to HX-30d CK3
		HX-30d-DS1 to HX-30d DS3
	45d	HX-45d-CK1 to HX-45d CK3
		HX-45d-DS1 to HX-45d DS3

Table S2. The effect of drought stress on the Relative Water content of *C. oleifera* petals.

Descriptives								
	**N**	**Mean**	**Std. Deviation**	**Std. Error**	**95% Confidence Interval for Mean**		**Minimum**	**Maximum**
					Lower Bound	Upper Bound		
15d CK	3	0.8574	0.0115	0.0066	0.8289	0.8858	0.8459	0.8688
15d DS	3	0.8373	0.0045	0.0026	0.8260	0.8485	0.8321	0.8406
30d CK	3	0.8568	0.0048	0.0028	0.8448	0.8688	0.8534	0.8623
30d DS	3	0.8270	0.0035	0.0020	0.8184	0.8356	0.8236	0.8305
45d CK	3	0.8652	0.0054	0.0031	0.8518	0.8786	0.8598	0.8706
45d DS	3	0.7977	0.0052	0.0030	0.7848	0.8106	0.7932	0.8034
Total	18	0.8402	0.0243	0.0057	0.8281	0.8523	0.7932	0.8706

Table S3. The effect of drought stress on the significant difference in Relative Water content among groups of *C. oleifera* petals.

		N	Subset for alpha = 0.05		
			1	2	3
**Duncan[a]**	**45d DS**	3	0.7977		
	30d DS	3		0.8270	
	15d DS	3		0.8373	
	30d CK	3			0.8568
	15d CK	3			0.8574
	45d CK	3			0.8652
	Sig.		1.000	0.07200	0.1480

Means for groups in homogeneous subsets are displayed.
a. Uses Harmonic Mean Sample Size = 3.000.

Table S4. The effect of drought stress on the Relative conductivity of *C. oleifera* petals.

Descriptives								
	**N**	**Mean**	**Std. Deviation**	**Std. Error**	**95% Confidence Interval for Mean**		**Minimum**	**Maximum**
					Lower Bound	Upper Bound		
15d CK	3	0.2217	0.0074	0.0043	0.2033	0.2400	0.2135	0.2279
15d DS	3	0.2375	0.0012	0.0007	0.2345	0.2405	0.2364	0.2388
30d CK	3	0.2206	0.0049	0.0028	0.2085	0.2327	0.2154	0.2251
30d DS	3	0.2431	0.0034	0.0020	0.2346	0.2517	0.2394	0.2462
45d CK	3	0.2241	0.0043	0.0025	0.2135	0.2346	0.2197	0.2282
45d DS	3	0.2536	0.0033	0.0019	0.2455	0.2618	0.2503	0.2569
Total	18	0.2334	0.0132	0.0031	0.2269	0.2400	0.2135	0.2569

Table S5. The effect of drought stress on the significant difference in Relative conductivity among groups of *C. oleifera* petals.

		**N**	**Subset for alpha = 0.05**		
			**1**	2	3
Duncan[a]	30d CK	3	0.2206		
	15d CK	3	0.2217		
	45d CK	3	0.2241		
	15d DS	3		0.2376	
	30d DS	3		0.2431	
	45d DS	3			0.2536
	Sig.		0.3860	0.1520	1.000

Means for groups in homogeneous subsets are displayed.
a. Uses Harmonic Mean Sample Size = 3.000.

Table S6. The effect of drought stress on the MDA content of *C. oleifera* petals.

Descriptives								
	**N**	**Mean**	**Std. Deviation**	**Std. Error**	**95% Confidence Interval for Mean**		**Minimum**	**Maximum**
					Lower Bound	Upper Bound		
HX15d CK	3	72.7400	4.2260	2.4399	62.2420	83.2380	69.2800	77.4500
HX30d DS	3	100.4367	4.9484	2.8570	88.1441	112.7292	97.1700	106.1300
HX45d CK	3	113.8800	9.9792	5.7615	89.0903	138.6697	102.3800	120.2600
HX15d DS	3	83.9233	1.2378	0.7147	80.8484	86.9983	82.5200	84.8600
HX30d DS	3	113.2700	4.8934	2.8252	101.1142	125.4258	110.3900	118.9200
HX45d DS	3	126.6967	7.0425	4.0660	109.2021	144.1912	119.1600	133.1100
Total	18	101.8244	19.7391	4.6526	92.0084	111.6405	69.2800	133.1100

Table S7. The effect of drought stress on the significant difference in MDA content among groups of *C. oleifera* petals.

		N	Subset for alpha = 0.05				
			1	2	3	4	5
Duncan[a]	HX15d CK	3	72.7400				
	HX15d DS	3		83.9233			
	HX30d DS	3			100.4367		
	HX30d DS	3				113.2700	
	HX45d CK	3				113.8800	
	HX45d DS	3					126.6967
	Sig.		1.000	1.000	1.000	0.9030	1.000

Means for groups in homogeneous subsets are displayed.
a. Uses Harmonic Mean Sample Size = 3.000.

Table S8. The effect of drought stress on the CAT activity in *C. oleifera* pistils and stamens.

Descriptives								
	N	Mean	Std. Deviation	Std. Error	95% Confidence Interval for Mean		Minimum	Maximum
					Lower Bound	Upper Bound		
15d CK	3	899.0000	16.8226	9.7125	857.2103	940.7897	886.0000	918.0000
30d CK	3	1003.3333	21.5019	12.4142	949.9196	1056.7471	982.0000	1025.0000
45d CK	3	1086.6667	13.6137	7.8599	1052.8483	1120.4850	1076.0000	1102.0000
15d DS	3	956.3333	24.5832	14.1931	895.2653	1017.4014	937.0000	984.0000
30d DS	3	995.0000	35.3836	20.4287	907.1022	1082.8978	963.0000	1033.0000
45d DS	3	1052.0000	9.6437	5.5678	1028.0438	1075.9562	1045.0000	1063.0000
Total	18	998.7222	65.4431	15.4251	966.1781	1031.2663	886.0000	1102.0000

Table S9. The effect of drought stress on the significant difference in CAT activity among groups of *C. oleifera* pistils and stamens.

		N	Subset for alpha = 0.05			
			1	2	3	4
Duncan[a]	15d CK	3	899.0000			
	15d DS	3		956.3333		
	30d DS	3		995.0000	995.0000	
	30d CK	3			1003.3333	
	45d DS	3				1052.0000
	45d CK	3				1086.6667
	Sig.		1.000	0.0520	0.6500	0.0770

Means for groups in homogeneous subsets are displayed.
a. Uses Harmonic Mean Sample Size = 3.000.

Table S10. The effect of drought stress on the POD activity in *C. oleifera* pistils and stamens.

Descriptives								
	**N**	**Mean**	**Std. Deviation**	**Std. Error**	**95% Confidence Interval for Mean**		**Minimum**	**Maximum**
					Lower Bound	Upper Bound		
15d CK	3	22,362.0000	496.0484	286.3937	21,129.7475	23,594.2525	21,842.0000	22,830.0000
30d CK	3	25,599.0000	1037.7895	599.1680	23,020.9880	28,177.0120	24,452.0000	26,473.0000
45d CK	3	30,337.6667	1509.9418	871.7653	26,586.7632	34,088.5701	29,352.0000	32,076.0000
15d DS	3	24,930.3333	97.6797	56.3954	24,687.6834	25,172.9833	24,821.0000	25,009.0000
30d DS	3	30,405.6667	565.2401	326.3415	29,001.5325	31,809.8008	29,890.0000	31,010.0000
45d DS	3	35,575.0000	2013.6581	1162.5861	30,572.7959	40,577.2041	33,250.0000	36,762.0000
Total	18	28,201.6111	4614.9615	1087.7569	25,906.6448	30,496.5775	21,842.0000	36,762.0000

Table S11. The effect of drought stress on the significant difference in POD activity among groups of *C. oleifera* pistils and stamens.

		N	Subset for alpha = 0.05			
			1	2	3	4
Duncan[a]	15d CK	3	22,362.0000			
	15d DS	3		24,930.3333		
	30d CK	3		25,599.0000		
	45d CK	3			30,337.6667	
	30d DS	3			30,405.6667	
	45d DS	3				35,575.0000
	Sig.		1.000	0.4910	0.9440	1.000

Means for groups in homogeneous subsets are displayed.
a. Uses Harmonic Mean Sample Size = 3.000.

Table S12. The effect of drought stress on the SOD activity in *C. oleifera* pistils and stamens.

Descriptives								
	**N**	**Mean**	**Std. Deviation**	**Std. Error**	**95% Confidence Interval for Mean**		**Minimum**	**Maximum**
					Lower Bound	Upper Bound		
HX45d DS	3	230.0000	10.59575	6.11746	203.6787	256.3213	217.8000	236.9000
00HX30d DS	3	165.4333	6.25247	3.60986	149.9013	180.9653	159.7000	172.1000
HX15d DS	3	145.7000	9.32577	5.38424	122.5335	168.8665	136.8000	155.4000
HX45d CK	3	182.8000	5.16430	2.98161	169.9712	195.6288	178.7000	188.6000
HX30d CK	3	152.6333	5.27857	3.04759	139.5206	165.7460	146.6000	156.4000
HX15d CK	2	119.3500	3.46482	2.45000	88.2198	150.4802	116.9000	121.8000
Total	17	168.7294	35.11721	8.51717	150.6738	186.7850	116.9000	236.9000

Table S13. The effect of drought stress on the significant difference in SOD activity among groups of *C. oleifera* pistils and stamens.

		N	Subset for alpha = 0.05				
			**1**	**2**	**3**	4	5
Duncan[a,b]	HX15d CK	2	119.3500				
	HX15d DS	3		145.7000			
	HX30d CK	3		152.6333	152.6333		
	HX30d DS	3			165.4333		
	HX45d CK	3				182.8000	
	HX45d DS	3					230.0000
	Sig.		1.000	0.2920	0.0660	1.000	1.000

Means for groups in homogeneous subsets are displayed.
a. Uses Harmonic Mean Sample Size = 2.769.

Table S14. The effect of drought stress on the SS content in *C. oleifera* pistils and stamens.

Descriptives

	N	Mean	Std. Deviation	Std. Error	95% Confidence Interval for Mean		Minimum	Maximum
					Lower Bound	Upper Bound		
15d CK	3	19.9500	0.62000	0.35796	18.4098	21.4902	19.4700	20.6500
30d CK	3	23.9067	0.49642	0.28661	22.6735	25.1398	23.3900	24.3800
45d CK	3	29.5667	0.65225	0.37658	27.9464	31.1870	29.0900	30.3100
15d DS	3	24.0633	0.28361	0.16374	23.3588	24.7679	23.8800	24.3900
30d DS	3	28.6100	0.98879	0.57088	26.1537	31.0663	27.5400	29.4900
45d DS	3	32.2000	0.85610	0.49427	30.0733	34.3267	31.5500	33.1700
Total	18	26.3828	4.27842	1.00843	24.2552	28.5104	19.4700	33.1700

Table S15. The effect of drought stress on the significant difference in SS content among groups of *C. oleifera* pistils and stamens.

		N	Subset for alpha = 0.05			
			**1**	**2**	3	4
Duncan[a]	15d CK	3	19.9500			
	30d CK	3		23.9067		
	15d DS	3		24.0633		
	30d DS	3			28.6100	
	45d CK	3			29.5667	
	45d DS	3				32.2000
	Sig.		1.000	0.7850	0.1150	1.000

Means for groups in homogeneous subsets are displayed.
a. Uses Harmonic Mean Sample Size = 3.000.

Table S16. The effect of drought stress on the SP content in *C. oleifera* pistils and stamens.

Descriptives								
	**N**	**Mean**	**Std. Deviation**	**Std. Error**	**95% Confidence Interval for Mean**		**Minimum**	**Maximum**
					Lower Bound	Upper Bound		
15d CK	3	2.2283	0.02532	0.01462	2.1654	2.2912	2.2000	2.2500
30d CK	3	3.2517	0.14550	0.08400	2.8902	3.6131	3.1200	3.4100
45d CK	3	4.1247	0.06734	0.03888	3.9574	4.2919	4.0500	4.1800
15d DS	3	3.2477	0.20115	0.11614	2.7480	3.7474	3.0800	3.4700
30d DS	3	4.3887	0.22715	0.13114	3.8244	4.9529	4.2200	4.6500
45d DS	3	4.9533	0.15153	0.08749	4.5769	5.3298	4.8600	5.1300
Total	18	3.6991	0.92905	0.21898	3.2370	4.1611	2.2000	5.1300

Table S17. The effect of drought stress on the significant difference in SP content among groups of *C. oleifera* pistils and stamens.

		**N**	**Subset for alpha = 0.05**			
			**1**	**2**	3	4
Duncan[a]	15d CK	3	2.2283			
	15d DS	3		3.2477		
	30d CK	3		3.2517		
	45d CK	3			4.1247	
	30d DS	3			4.3887	
	45d DS	3				4.9533
	Sig.		1.000	0.9750	0.0570	1.000

Means for groups in homogeneous subsets are displayed.
a. Uses Harmonic Mean Sample Size = 3.000.

Table S18. The effect of drought stress on the Pro content in *C. oleifera* pistils and stamens.

Descriptives								
	**N**	**Mean**	**Std. Deviation**	**Std. Error**	**95% Confidence Interval for Mean**		**Minimum**	**Maximum**
					Lower Bound	Upper Bound		
15d CK	3	56.4333	1.7411	1.0052	52.1082	60.7585	54.4500	57.7100
30d CK	3	69.7667	2.5007	1.4438	63.5545	75.9788	67.1200	72.0900
45d CK	3	89.3067	2.1200	1.2240	84.0403	94.5731	87.1900	91.4300
15d DS	3	70.1967	0.7586	0.4380	68.3123	72.0811	69.3300	70.7400
30d DS	3	88.2500	4.0146	2.3178	78.2773	98.2227	83.6700	91.1600
45d DS	3	97.1033	4.7045	2.7161	85.4168	108.7899	92.7000	102.0600
Total	18	78.5094	14.7039	3.4657	71.1974	85.8215	54.4500	102.0600

Table S19. The effect of drought stress on the significant difference in Pro content among groups of *C. oleifera* pistils and stamens.

		N	Subset for alpha = 0.05			
			**1**	**2**	3	4
Duncan[a]	15d CK	3	56.4333			
	30d CK	3		69.7667		
	15d DS	3		70.1967		
	30d DS	3			88.2500	
	45d CK	3			89.3067	
	45d DS	3				97.1033
	Sig.		1.000	0.8620	0.6700	1.000

Means for groups in homogeneous subsets are displayed.
a. Uses Harmonic Mean Sample Size = 3.000.

Table S20. The effect of drought stress on the ABA content in *C. oleifera* pistils and stamens.

Descriptives								
	**N**	**Mean**	**Std. Deviation**	**Std. Error**	**95% Confidence Interval for Mean**		**Minimum**	**Maximum**
					Lower Bound	Upper Bound		
15d CK	3	131.9600	3.5514	2.0504	123.1378	140.7822	129.8400	136.0600
15d DS	3	388.2700	4.4878	2.5910	377.1217	399.4183	383.3600	392.1600
30d CK	3	68.3900	0.8489	0.4901	66.2811	70.4989	67.4100	68.9000
30d DS	3	474.8233	9.0475	5.2236	452.3481	497.2985	467.7900	485.0300
45d CK	3	51.6533	1.1927	0.6886	48.6906	54.6161	50.7700	53.0100
45d DS	3	211.4833	2.7328	1.5778	204.6948	218.2719	208.3800	213.5300
Total	18	221.0967	164.0347	38.6633	139.5242	302.6692	50.7700	485.0300

Table S21. The effect of drought stress on the significant difference in ABA content among groups of *C. oleifera* pistils and stamens.

		N	Subset for alpha = 0.05					
			**1**	**2**	**3**	**4**	5	6
Duncan[a]	45d CK	3	51.6533					
	30d CK	3		68.3900				
	15d CK	3			131.9600			
	45d DS	3				211.4833		
	15d DS	3					388.2700	
	30d DS	3						474.8233
	Sig.		1.000	1.000	1.000	1.000	1.000	1.000

Means for groups in homogeneous subsets are displayed.
a. Uses Harmonic Mean Sample Size = 3.000.

Table S22. The effect of drought stress on the GA_3 content in *C. oleifera* pistils and stamens.

Descriptives								
	N	Mean	Std. Deviation	Std. Error	95% Confidence Interval for Mean		Minimum	Maximum
					Lower Bound	Upper Bound		
15d CK	3	0.1160	0.01709	0.0099	0.0736	0.1584	0.1000	0.1300
15d DS	3	0.3433	0.02369	0.0137	0.2845	0.4022	0.3200	0.3600
30d CK	3	0.1653	0.00751	0.0043	0.1467	0.1840	0.1600	0.1700
30d DS	3	0.5203	0.03017	0.0174	0.4454	0.5953	0.5000	0.5600
45d CK	3	0.1297	0.01721	0.0099	0.0869	0.1724	0.1100	0.1400
45d DS	3	0.4383	0.03623	0.0209	0.3483	0.5283	0.4000	0.4700
Total	18	0.2855	0.16356	0.0386	0.2042	0.3668	0.1000	0.5600

Table S23. The effect of drought stress on the significant difference in GA_3 content among groups of *C. oleifera* pistils and stamens.

		N	Subset for alpha = 0.05				
			1	2	3	4	5
Duncan[a]	15d CK	3	0.1160				
	45d CK	3	0.1297	0.1297			
	30d CK	3		0.1653			
	15d DS	3			0.3433		
	45d DS	3				0.4383	
	30d DS	3					0.5203
	Sig.		0.4970	0.0930	1.000	1.000	1.000

Means for groups in homogeneous subsets are displayed.
a. Uses Harmonic Mean Sample Size = 3.000.

Table S24. The effect of drought stress on the IAA content in *C. oleifera* pistils and stamens.

Descriptives								
	N	Mean	Std. Deviation	Std. Error	95% Confidence Interval for Mean		Minimum	Maximum
					Lower Bound	Upper Bound		
15d CK	3	0.9510	0.1431	0.0826	0.5954	1.3066	0.8220	1.1050
15d DS	3	1.0913	0.0356	0.0205	1.0030	1.1797	1.0570	1.1280
30d CK	3	1.1677	0.1697	0.0980	0.7461	1.5892	1.0030	1.3420
30d DS	3	1.9737	0.1123	0.0648	1.6948	2.2526	1.8460	2.0570
45d CK	3	1.4533	0.0936	0.0540	1.2209	1.6858	1.3810	1.5590
45d DS	3	2.5373	0.2944	0.1700	1.8060	3.2687	2.2610	2.8470
Total	18	1.5291	0.5914	0.1394	1.2349	1.8232	0.8220	2.8470

Table S25. The effect of drought stress on the significant difference in IAA content among groups of *C. oleifera* pistils and stamens.

		N	**Subset for alpha = 0.05**			
			**1**	**2**	3	4
Duncan[a]	15d CK	3	0.9510			
	15d DS	3	1.0913			
	30d CK	3	1.1677	1.1677		
	45d CK	3		1.4533		
	30d DS	3			1.9737	
	45d DS	3				2.5373
	Sig.		0.1460	0.0520	1.000	1.000

Means for groups in homogeneous subsets are displayed.
a. Uses Harmonic Mean Sample Size = 3.000.

Table S26. The effect of drought stress on the CTK content in *C. oleifera* pistils and stamens.

Descriptives

	**N**	**Mean**	**Std. Deviation**	**Std. Error**	**95% Confidence Interval for Mean**		**Minimum**	**Maximum**
					Lower Bound	Upper Bound		
15d CK	3	2.1317	0.2110	0.1218	1.6076	2.6557	1.9000	2.3000
15d DS	3	2.0750	0.0880	0.0508	1.8564	2.2936	1.9800	2.1600
30d CK	3	2.9417	0.1623	0.0937	2.5385	3.3448	2.7700	3.0900
30d DS	3	2.0673	0.0860	0.0497	1.8536	2.2810	2.0100	2.1700
45d CK	3	1.4897	0.1032	0.0596	1.2334	1.7459	1.3900	1.6000
45d DS	3	5.4937	0.2865	0.1654	4.7819	6.2055	5.1700	5.6900
Total	18	2.6998	1.3652	0.3218	2.0209	3.3787	1.3900	5.6900

Table S27. The effect of drought stress on the significant difference in CTK content among groups of *C. oleifera* pistils and stamens.

		N	Subset for alpha = 0.05			
			1	2	3	4
Duncan[a]	45d CK	3	1.4897			
	30d DS	3		2.0673		
	15d DS	3		2.0750		
	15d CK	3		2.1317		
	30d CK	3			2.9417	
	45d DS	3				5.4937
	Sig.		1.000	0.6720	1.000	1.000

Means for groups in homogeneous subsets are displayed.
a. Uses Harmonic Mean Sample Size = 3.000.

Table S28. The effect of drought stress on the SA content in *C. oleifera* pistils and stamens.

Descriptives								
	N	Mean	Std. Deviation	Std. Error	95% Confidence Interval for Mean		Minimum	Maximum
					Lower Bound	Upper Bound		
15d CK	3	1490.7333	21.01650	12.13388	1438.5254	1542.9412	1478.40	1515.00
15d DS	3	1844.1667	15.20077	8.77617	1806.4059	1881.9275	1826.70	1854.40
30d CK	3	1379.6333	15.05468	8.69182	1342.2354	1417.0312	1364.80	1394.90
30d DS	3	1449.2667	5.57524	3.21887	1435.4170	1463.1163	1445.10	1455.60
45d CK	3	609.4333	7.78224	4.49308	590.1012	628.7655	602.80	618.00
45d DS	3	643.9000	7.12671	4.11461	626.1963	661.6037	635.70	648.60
Total	18	1236.1889	468.67740	110.46832	1003.1211	1469.2567	602.80	1854.40

Table S29. The effect of drought stress on the significant difference in SA content among groups of *C. oleifera* pistils and stamens.

		N	Subset for alpha = 0.05					
			1	2	3	4	5	6
Duncan[a]	45d CK	3	609.4333					
	45d DS	3		643.9000				
	30d CK	3			1379.6333			
	30d DS	3				1449.2667		
	15d CK	3					1490.7333	
	15d DS	3						1844.1667
	Sig.		1.000	1.000	1.000	1.000	1.000	1.000

Means for groups in homogeneous subsets are displayed.
a. Uses Harmonic Mean Sample Size = 3.000.

Table S30. The effect of drought stress on the JA content in *C. oleifera* pistils and stamens.

Descriptives								
	N	Mean	Std. Deviation	Std. Error	95% Confidence Interval for Mean		Minimum	Maximum
					Lower Bound	Upper Bound		
15d CK	3	15.1240	0.4868	0.2811	13.9146	16.3334	14.6670	15.6360
15d DS	3	17.3020	0.5689	0.3284	15.8888	18.7152	16.7030	17.8350
30d CK	3	7.2870	0.2578	0.1489	6.6465	7.9275	7.0100	7.5200
30d DS	3	4.1243	0.1065	0.0615	3.8599	4.3888	4.0020	4.1960
45d CK	3	2.4610	0.0265	0.0153	2.3951	2.5269	2.4340	2.4870
45d DS	3	0.6290	0.1004	0.0579	0.3797	0.8783	0.5150	0.7040
Total	18	7.8212	6.4816	1.5277	4.5980	11.0444	0.5150	17.8350

Table S31. The effect of drought stress on the significant difference in JA content among groups of *C. oleifera* pistils and stamens.

		N	Subset for alpha = 0.05					
			**1**	**2**	**3**	**4**	5	6
Duncan[a]	45d DS	3	0.6290					
	45d CK	3		2.4610				
	30d DS	3			4.1243			
	30d CK	3				7.2870		
	15d CK	3					15.1240	
	15d DS	3						17.3020
	Sig.		1.000	1.000	1.000	1.000	1.000	1.000

Means for groups in homogeneous subsets are displayed.
a. Uses Harmonic Mean Sample Size = 3.000.
